# 150th Anniversary of global adenoid investigations: unanswered questions and unsolved problems

**DOI:** 10.3389/fped.2023.1179218

**Published:** 2023-07-14

**Authors:** Aleksander Zwierz, Krystyna Masna, Krzysztof Domagalski, Paweł Burduk

**Affiliations:** ^1^ Department of Otolaryngology, Phoniatrics and Audiology, Faculty of Health Sciences, Ludwik Rydygier Collegium Medicum, Nicolaus Copernicus University, Bydgoszcz, Poland; ^2^ Department of Immunology, Faculty of Biological and Veterinary Sciences, Nicolaus Copernicus University, Torun, Poland

**Keywords:** adenoid hypertrophy, adenoid hypertrophy treatment, conservative treatment, AH, adenoid surgery

## Abstract

Although the problem of adenoid hypertrophy (AH) has been diagnosed and treated by doctors and scientists from around the world for the last 150 years, there is still no consensus regarding appropriate diagnosis, conservative treatment options, and qualification for surgery. This manuscript presents current knowledge on these issues and compares diagnostic methods and the effectiveness of treatment options. Factors that may influence the obtained treatment results are also described, and a questionnaire is proposed to compare the results of treatment. The objective of drawing attention to this problem is to obtain better results from conservative treatment in the future and better-qualified patients for surgical treatment.

## Introduction

**Figure 1:**
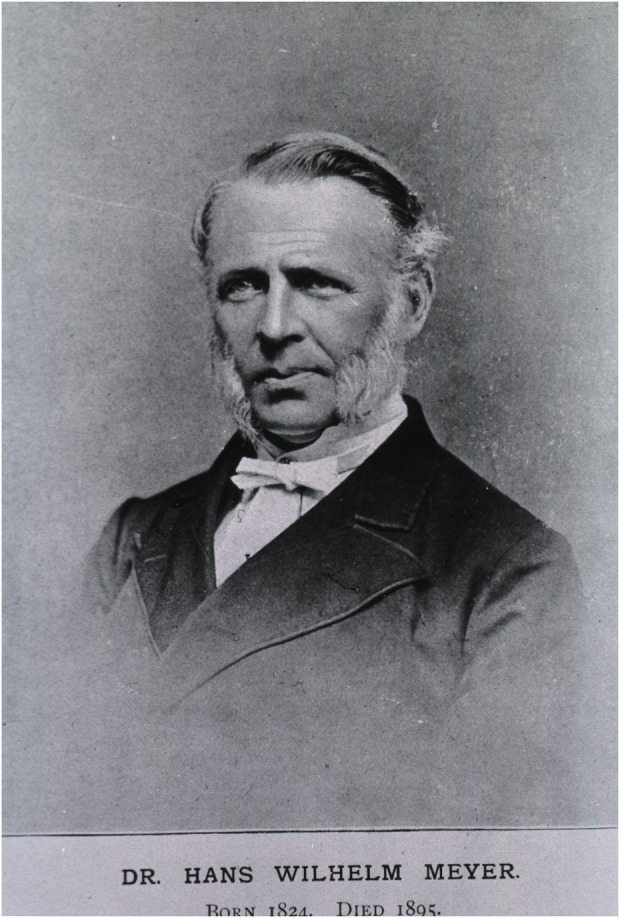
Hans Wilhelm Meyer.

Although the pharyngeal tonsil was first discovered and described by Conrad Victor Schneider in 1,661 as a prominent nasopharynx structure, it was only 150 years ago, in 1873, that the medical world started to concentrate its attention on this important field of children's disease (1, 2). Such interest was motivated by Hans Wilhelm Meyer's second publication, “Ueber adenoide Vegetationen in der Nasenrachenhohle,” in Arch. f. Ohrenheilkunde 1873, T-11 Bd. S. 211. VIII B., S. 120 and 241. Meyer is the father of the term “adenoid” and published the first report on an adenoid surgical resection in 1868 (Figure 1). However, although this first scientific report was significant, it attracted little attention. What would be the breakthrough manuscript was published by Wilhelm Meyer five years later, when he described his observations and experience in the surgical treatment of adenoids (2). From that time on, the medical world started paying more attention to this important field of disease. Specifically, the role and function of this nasopharyngeal structure in the pathogenesis of recurrent upper respiratory tract infection and rhinorrhoea began to be investigated by doctors. Adenoid hypertrophy (AH) was also found to be related to otitis media with effusion (3). Moreover, the concept of a “united airway disease” suggested that AH and rhinitis may have impact on the lower respiratory tract, which was confirmed in children suffering from asthma (4).

AH is one of the most common disorders in children. All associated indications, such as mouth breathing, snoring, nasal blockage, chronic rhinitis, and nasal speech are called “adenoid symptoms”. The consequent obstruction of the nose may also cause recurrent sinusitis, asthma, sleep apnoea, and otitis media with effusion. In some cases, AH may cause serious health deterioration and impair child development (5). For instance, Bitar et al. showed that 57.7% of young children suffering from nasal blockage and admitted to ENT outpatient clinics had AH (6).

### Diagnosis

#### What is the best method for diagnosing adenoid hypertrophy?

The father of adenoid diagnostics, Hans Wilhelm Meyer, used his finger to explore the patient's mouth to confirm hypertrophy. He described his own methods of diagnosis in 1868 and was the first to perform resection surgery with an adenotome (7). Since then, many diagnostic methods have been introduced in the search for the most accurate approach, as well as the one that is most comfortable and least burdensome for patients, most of whom are young, not well cooperative children. Invasive techniques and imaging technology were later implemented. For the first group of diagnostic techniques, physical examinations were performed through the mouth or nose. For many years, doctors would palpate the adenoids with a finger then use the less traumatic approach of transoral mirror examination. The development of rigid and later flexible nasopharyngoscopies allowed for a pharyngeal examination to be performed through the nose. Thinner rigid nasopharyngeal endoscopy (RNE) and flexible nasopharyngeal endoscopy (FNE) became common methods for nasopharyngeal examination. Additionally, video fluoroscopy and acoustic rynomanometry for nasal diagnosis were developed. The second diagnostic tool group consisted of lateral x-ray of the nasopharynx (lateral cephalogram), ultrasonography, computer tomography (CT), and magnetic resonance imaging (MRI) (8–12).

The results of these initial tests were often based on the doctors' own experiences of feeling what is often immeasurable. Other results are based on measurable parameters, which do not always have to be related only to AH. In some cases, the condition may be simulated by other reasons (i.e., thermal seasons). Other factors related to nose and nasopharyngeal obstruction include nasal concha hypertrophy, nasal septal deviation, polyps, and allergic rhinitis (9, 13). The presence of such conditions can make it difficult to objectify and compare the results. Newly introduced diagnostic methods should still be compared to the results of transoral mirror examination or nasal endoscopy to ensure effectiveness (9, 12–14). However, this only occurs in some cases. Furthermore, in selected cases, the results of the intraoperative mirror exam may not correlate with preoperative FNE. Such circumstances may arise in children with small- and medium-sized adenoid hypertrophy (A/C ratio beneath 75%) (9). Moreover, Patel suggests that intraoperative mirror examination performed in a horizontal position in anaesthesia with relaxation may also be fraught with observation errors (9).

The sensitivity and specificity of lateral x-rays of the nasopharynx (lateral cephalometry) have reached 61%–75% and 41%–96% respectively (10, 15). More objective results are achieved when the diagnostician takes an x-ray picture at the end of patient's inspiration phase. This is especially difficult in the case of young, non-cooperating, and often frightened children (16). According to Major et al., the size of the adenoid is often overestimated in lateral cephalometry. As a result, lateral x-rays are useful for measuring the free airway space between the adenoid and soft palate (15). This is due to the fact that the diagnostic results of lateral cephalometry are shown in a single two-dimensional summation picture (15–17). As an alternative, lateral cephalometric radiographs are a simple, non-expensive, sufficiently informative method. Moreover, new digital x-ray apparatuses decrease radiation expousure (15, 17). While Mlynarek at al. did not find a correlation between lateral cephalometry and obstructive symptoms scores (OSS), a relationship has been identified between FNE and OSS (18). However, in another study, Caylakli found a correlation between the results of lateral cephalometry and those of FNE (19).

The high radiation doses of other available imaging methods, such as computer tomography (CT), cone bean computer tomography (CBCT), and time consuming such as magnetic resonance imaging (MRI) exclude these methods from being used repeatedly (20). From that reasons promising seems to be ultrasonography of the adenoid tissue. Wang et al. tried to assess AH with ultrasonography, and despite the encouraging results, further evaluation is needed (21).

A definite advantage of invasive diagnostics, in addition to the static assessment of anatomical structures, is their ability to obtain dynamic information on the functioning of the nose and nasopharynx. In these examinations, a colour image is obtained, which allows for differentiation between the physiological and inflammatory conditions of the mucous membrane, as characterised by the type of mucous in the nose and its coverage of the adenoids.

Invasive nasopharyngeal diagnostics may cause discomfort and pain. In the absence of the child's cooperation, general anaesthesia is required, but such circumstances are rare for an experienced paediatric laryngologist. According to Ysunza et al., video fluoroscopy of the nasopharynx shows high sensitivity (100%) and specificity (90%). Unfortunately, this diagnostic tool produces a 260 micro-sievert irradiation dose (10, 15). Flexible nasopharyngoscopy is less traumatic than rigid endoscopy, and mentioned above, it may be performed without anaesthesia and provide important information about the nose, nasopharynx, and adenoid state (22, 23). In the context of COVID-19 tests, in the patients' opinions, this examination is less painful than testing with a COVID-19 nasal-swab. The sensitivity and specificity of flexible nasopharyngoscopy in the assessment of AH have reached 97.3% and 72.7%, respectivly (24). Today, flexible endoscopic examination should be the gold standard in AH examination and serve as a reference test for newly introduced diagnostic methods.

#### What should be appreciated for adenoid hypertrophy classification?

**Figure 2:**
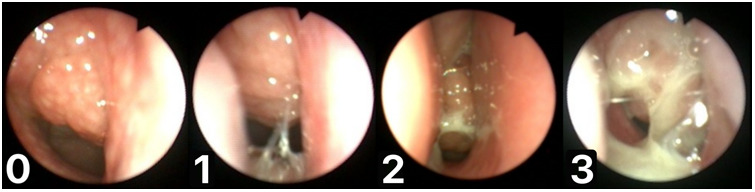
Mucus of adenoid scale by nasopharyngoscopy assessment (MASNA), 0—no mucus, 1—residue of clear watery mucus, 2—some amount of dense mucus, 3—copious thick dense mucus.

In clinical adenoid evaluation in children, the percentage of nasopharyngeal space occupied by the adenoid is most often used for adenoid size assessment. This is referred to as the adenoid-to-choana scale or ratio (A/C scale, A/C ratio) and is usually measured with an accuracy of up to 5%. For better assessment of patient groups, different anatomical and clinical classifications are used. In fact, many authors have introduced their own AH classifications. Although they are often similar, each may contain its own modified concept of the anatomical assessment of nasopharyngeal structures in relation to a particular clinical condition. The most common classification has been proposed by Cassano and is based on a four-step pictorial scheme describing the occupation of the nasopharynx by the adenoid (I° - 0%–25%, II° - 26%–50%, III° - 51%–75%, or IV° - 76%–100%) (25). In another five-step scale introduced by Zalzal, 0° indicates 0% obstruction of the choanae,1° less than 40% obstruction, 2° 41%–70% obstruction, 3° 71%–90% obstruction, and 4° complete obstruction (91%–100%), with adenoid tissue touching the relaxed soft palate (26). Another three-degree classification of AH was proposed by Parikh and Bolesławska (27, 28). Some of these classifications also differentiate relations between the adenoid and eustachian tube (27, 29). Flexible endoscopic adenoid investigation also allows for the classification of mucous coverage of the adenoid. For these reasons, the Mucus of Adenoid Scale by Nasopharyngoscopy Assessment (MASNA) was proposed. This four-point classification scheme accounts for the amount of mucus covering the adenoid (0° corresponds to no mucus, 1° describes the residue of clear watery mucus, 2° indicates some amount of dense mucus, and 3° indicates copious, thick, dense mucus; Figure 2) (23). In light of many proposed and used scales, there is still an important question that remains: what degree of AH is indicative of a large adenoid which should be surgically removed? This would help unify the results and facilitate further analysis.

#### What does it mean for an adenoid to be large?

In our daily practice, we often encounter patients who have previously been examined by other doctors who, based on anterior rhinoscopy and symptoms reported by parents, declare that the adenoid is large and suggest its removal. Unfortunately, these statements often do not correlate with endoscopic examination of the nasopharynx. Our intraoperative comparison of adenoid size with preoperative endoscopic adenoid assessment indicated that a 75% A/C ratio or more is equivalent to an intraoperatively removed large adenoid (24). For this reason, the classification proposed by Cassano seems to be more adequate for AH assessment because the degree of AH in this scale is equal to a large adenoid, and, in this case, adenoidectomy should be considered (25).

#### Does the adenoid involute with age? Is it worth waiting for an adenoidectomy?

For the most part, knowledge about adenoid tissue involution is based on Scammons's theory, which is over 100 years old, and ENT doctors' own experiences in treating children (30). According to Scammon's curves, adenoid tissue grows during childhood, leading to involution in adulthood (30). Still, there is a general lack of longitudinal observational studies on adenoid development in children, and only three are based on lateral nasopharynx x-rays studies. As shown above, lateral cephalometry may overestimate adenoid size and should be used specifically for measuring the narrowest airway space between the nose and the nasopharynx (15, 17, 31, 32). Our study showed that the involution of the adenoid proceeds slowly (24). Endoscopic examinations in the analysed group of preschool children indicated that in only 7.9% of the children, adenoid size changed by more than 15% on the A/C scale after one year of observation. In 21.6% of children, this change occurred over a period of two years, and over a period of three years in 45%. These findings are similar to the results of the longitudinal lateral cephalometric studies performed by Yamada, which showed that an overgrowth of adenoids appeared in preschool children, but there were no significant changes in the adenoid size at 8–12 years age (32). The growth and development patterns of nasopharynx lymphoid tissues are different for each patient. In our opinion, there is still a need for an accurate and broad analysis of adenoid involution in children with the use of objective adenoid size assessment.

### Treatment

#### What is an adequate conservative medical treatment for adenoid hypertrophy?

Various methods of conservative treatment of AH have been used thus far. Their results are often difficult to evaluate because many studies have not undertaken a classic assessment of the size of the adenoid or its mucus coverage, instead only analysing the reduction of ailments and adenoid symptoms or, for example, performing a spirometry test. Therefore, the maximal conservative medical treatment of AH and how long it should last is still not known.

##### Intranasal topical steroids

Intranasal steroid treatment has been the most common treatment for AH and related symptoms for many years. Numerous studies have confirmed the beneficial effect of topical steroids on complaints related to AH or for decreases in adenoid symptoms (Table 1) (33–46). Significantly less publications refer to the objective postoperative assessment of adenoid size. According to Jazi at al., adenoid tissue regression after steroid treatment in FNE examination was less significant than what would be considered clinical improvement (44). However, *in vitro* clinical trials showed some impact of corticosteroids on reducing adenoid tissue proliferation (47). These effects were confirmed by the identification of the GCR-α and GCR-β human glucocorticoid receptors in adenoid tissue (48). These two receptors are ligands for glucocorticoid and regulate the tissue response for steroids (48). It should be emphasized that there is a lack of studies on the long-term outcomes of AH treatment with nasal steroids on adenoid size in children. Almost all the existing research has analysed changes of adenoid symptoms or measured adenoid size (A/C ratio) immediately after conservative treatment and not after a leeway period without the usage of topical steroids. The reports presented by Criscuoli are staggering, indicating that 70% children undergo adenoidectomy in the two-year follow-up period after treatment, despite the fact that immediately after treatment, 45% of children achieve significant improvement in their symptoms (43). Our long-term results 3–6 months after the discontinuation of medication did not indicate an adenoid size change and suggest that the therapy should be used continuously for a long-term period. A low rate of side effects allows for these steroids to be used topically for a long period of time (43, 46, 49, 50). This tendency is especially visible in recent works, where the period of use of nasal steroids has been extended (42, 43).

**Table 1:** Research on the effect of topical steroids on adenoid size or symptoms.

Author/year/country	Age	Number of patients treated with steroids	Medication	Time of treatment	Time of final results counting	Main results
Cengel (33) 2005 Turkey	3–15	122	MF	6 weeks	at the end of therapy	improvement of OME in 42.2% of children
Ciprandi (34) 2007 Italy	3–6	139	F	8 weeks	at the end of therapy	reduction of A/C ratio in 72% patients
Demirhan (35) 2010 Turkey	4–16	25	MF	8 weeks	at the end of therapy	symptoms improvement, adenoidectomy was not necessary in 76% of children
Mohebi (36) 2014 Iran	2–11 (2–4 and 5–11)	51	MF	3 months	at the end of therapy	improvement
Gupta (37) 2015 India	4–12	55	MF	4 weeks	at the end of therapy	improvement
Monga (38) 2020 India	3–11	30	MF	8 weeks	at the end of therapy	improvement
Rezende (39) 2015 Brazil	4–8	55	MF	6 weeks	at the end of therapy	decrease of adenoid size
Hassanzadeh (40) 2016 Iran	4–12	20	MF	4 weeks	at the end of therapy	decrease of adenoid size
Lepcha (41) 2002 India	3–12	13	B	8 weeks	at the end of therapy	**no improvement**
Berlucchi (42) 2008 Italy	3–7	21	MF	1–3 months before surgery or 15–31 months (mean 23) (2 weeks every month, suspended during the summer)	Before surgery (9 children) or at the end of the maintenance therapy (12 children)	Long-time MF therapy may obtain successful results
Criscuoli (43) 2003 Italy	Mean 3,8	53	B	26 weeks	24,52,100 weeks after treatment	relevant clinical improvement in 45% children immediately after therapy, finally, after 2 years 70% children performed surgery
Jazi (44) 2011 Iran	2–10	20	MF	6 weeks	1 and 8 weeks after treatment	adenoid regression in FNE was less significant than clinical improvement
Bhargava (45) 2014 India	2–12	100	MF	24 weeks	24 weeks after treatment	clinical improvement
Zwierz (46) 2022 Poland	3–6	165	MF	3 months	3 to 6 months after end of the treatment	no long-time effect of intranasal mometasone furoate on adenoid size, its mucus

MF, mometasone fluroate; F, flunisolide; B, beclomethasone.

##### Antihistamine drug therapies

It is estimated that 20%–40% of children worldwide are affected by allergic rhinitis (AR) (51, 52). A study performed by Eren et al. showed that skin prick tests were positive in 65.2% of young patients with AH symptoms (53). The prevalence of AH has been increased in children with allergies, which means that this treatment could only be effective in this group of patients (54). However, there is a discrepancy in age predominance in children diagnosed with AH and AR. AH is diagnosed between 3 and 4 years of age, whereas AR is usually diagnosed in children 6–7 years old. Moreover, the remaining group of patients with nonallergic rhinitis was not homogenous. These cases included local allergic, drug-induced, gustatory, atrophic, occupational, hormonal, cold-air induced, and idiopathic rhinitis. It seems that in this nonallergic rhinitis group, the most common form is local allergic rhinitis (LAR). The incidence of LAR in children ranges from 3.7% to 66.6% (55). LAR seems to be an underdiagnosed entity and not considered for the doctors.

Both allergic and nonallergic rhinitis are cases of chronic rhinitis characterised by the presence of inflammatory cells that act on the nasal mucosa. Activation of the mast cells in nonallergic rhinitis cause histamine and a variety of other mediators (e.g., eosinophil chemotactic factor of anaphylaxis, PAF, leukotrienes, and prostaglandins) to release that exacerbate the inflammatory reaction (56). The release of histamine also acts chemotactically on neutrophils. Since a significant group of children with AH may be affected by both AR and LAR, local or systemic antihistamine treatment may be initiated in the case of strong symptoms, such as sneezing, rhinorrhoea, congestion, and nasal itching. The use of antihistamine therapy in patients with adenoid symptoms may be considered and should be further investigated. This treatment could be applied in all AH patients and continued for patients whose adenoid symptoms decrease after initial treatment.

##### Hypertonic saline solutions

This type of solution is used as an auxiliary and has been shown to be highly effective in cleansing the nasal mucosa of residual secretions and allergens. Hypertonic solutions are more effective in this respect; it is also important that the effectiveness in cleansing the mucosa increases with the volume of solution used (57). Such solutions should be used as a supportive treatment for adenoid symptoms.

##### Halotherapy

The release of micronized medical iodized sodium chloride in indoor climate-controlled conditions is another option for AH treatment. A study performed by Gelardi showed higher percentages of adenotonsillar tissue reduction in children after 10 daily sessions of micronized salt inhalation in a salt room when compared to placebo (58). This result was not statistically significant, however, and the authors concluded that a large sample of patients would be needed to show statistically significant rates of adenoid reduction.

##### Adrenomimetic agents

Although one study has shown the supporting effect of using oxymetazoline in AH treatment with nasal steroids, the lack of symptoms of rhinitis medicamentosa, and the rebound effect on the mucous membrane, most authors do not recommend their chronic use due to increasing rebound nasal congestion (50, 59).

##### Antibiotics

Even in the latest research by Zuo et al., it has been shown that the adenoid is a habitat for aerobic bacteria that can affect the development of AH, and the associated symptoms and appropriate antimicrobial therapy seem to be obvious (60). In the past, several weeks of antibiotic therapy have been used to treat AH, but this method was discontinued due to the negative impact of systemic antibiotic use on the entire microbiome of a child's body (44, 61). Currently, the local supplementation of bacteria to modify the nasal and nasopharyngeal microbiomes is more popular (62). Another approach is a 12-week treatment with OM85-BV, which may improve the Th1 immune response by weakening the local inflammatory response in the adenoids (63).

##### Proton pump inhibitors

Some studies have indicated a correlation between AH and gastroesophageal reflux, or AH and laryngo-pharyngeal reflux (64–67). Sagar demonstrated that adenotonsillectomy resulted in complete resolution of GER in 80% of children and improvement in 20% (66). However, in contrast, Iqbal's did not support the efficacy of PPIs for adenoid hypertrophy in children (68).

##### Anti-leukotriene therapies

Recently, some publications have pointed to the effectiveness of treating obstructive sleep apnea (OSA) with anti-leukotriene drugs for three months and adenoid hypertrophy (Table 2) (69–73). Ras showed better outcomes for oral montelukast with intranasal steroid in the treatment of AH than single-use mometasone (69). This is consistent with Tuhanoglu et al.'s findings, who described better symptom recovery in children treated with combined montelucast and mometasone furoate therapy; however, their objective assessment of adenoid size measured by lateral cephalometry showed no difference between this group and groups treated with mometasonel or montelucast alone (70). A study performed by Goldbart et al. showed that the adenotonsilar tissue of children with OSA contained higher leukotriene levels than that with infectious tonsilitis, and for this reason, this anti-leukotriene therapy should be applied to treat children with OSA symptoms rather than infectious adenoid symptoms (74). Montelukast is not approved for the treatment of AH and AR in Europe. Serious side effects, including hyperactive sleep disorders and depression, should be taken into consideration if anti-leukotriene therapy is to be applied (75, 76). Perhaps for this reason, all therapeutic regimens administered so far have lasted no longer than three months (Table 2).

**Table 2:** Review of similar studies comparing effectiveness of anti-leukotriene therapy in children with adenoid hypertrophy. M: montelukast; MF: mometasone furoate.

Author year country	Age	Number of patients treated with montelucast/Control group	Medication	Time of treatment	Time of final result count	Main results
Ras (69) 2020 Egipt	3–10	50/50	M+MF Control MF	3 months	At the end of therapy, 3 months after treatment	Endoscopic A/C ratio examination Significantly better improvement over controls
Tuhanoglu (70) 2017 Turkey	4–10	30/30/30/30	M MF M+MF Placebo	3 months	At the end of therapy	Lateral cephalograms: Similar adenoid to air passage improvement in all groups, except placebo Best recovery in symptoms score in combined group
Shokouhi (71) 2015 Iran	4–12	30/30	M Placebo	3 months	At the end of therapy	Lateral cephalograms: Reduction of more than 25% in adenoid size in 76% treated patients vs. control (3.3%) Nasal endoscopy findings: Significant difference between groups after treatment
Liu (72) 2017 China	–	69/69	M+MF MF		At the end of therapy	M+MF more effectively reduced the adenoid nasopharynx ratio
Goldbart (73) 2012 Israel	2–10	23/23	M Placebo	3 months	At the end of the study	Lateral cephalogram radiography Decrease of the nasopharyngeal ratio in group treated with montelukast

**Figure 3:**
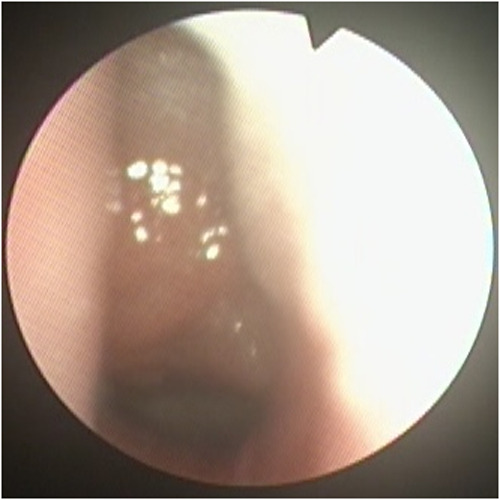
Endoscopic view of adenoid in 8-year-old boy treated with montelukast.

Our single observations of patients treated with juvenile asthma with the use of leukotriene for a few years did not confirm its role in decreasing adenoid size. Figure 3 shows an endoscopic view of an adenoid 8-year-old boy treated with 5 mg montelukast per day because of asthma for 3 years. Still, the role of anti-leukotriene therapies in decreasing adenoid size should be investigated.

In analysing the methods of conservative treatment described in the literature, there are still no spectacular effects or breakthroughs to be found. However, accurate diagnosis and clinical analysis should allow for the selection of patients who may be susceptible to medical treatment and able to avoid surgical treatment and related complications. Topical steroid treatment and saline irrigation should be applied before consideration of surgical treatment. Furthermore, antihistaminic drug and anti-leukotriene therapy studies should be analysed to evaluate possible benefits and side effects. Conservative treatment may be more effective with an A/C ratio beneath <75% (1–3 degrees of adenoid hypertrophy on the Cassano scale).

#### Is there an alternative treatment?

Some Chinese studies have indicated the efficacy of traditional Chinese herbal medicine for AH treatment in children, which showed better outcomes than Western medicine results (77–79). Zhao showed that oral Xiao-xian decoction combined with acupuncture (acupoint application) improved clinical symptoms of adenoid hypertrophy and may be suitable for long-term treatment (79).

However, there is still a variety of herbs mixtures used and no consensus on the treatment methods, as well as a lack of objectively evaluated measurements (80). Therefore, there is a need for long-term prospective clinical trials and a necessity to carry out evidence evaluation on the treatment of AH with Chinese or Western medicine to provide feasible and effective treatment options for clinics (78).

##### Acupuncture

Similar to Zaho's reports, the case presented by Deng showed the effectiveness of sphenopalatine ganglion electroacupuncture in widening the patency of the nasopharyngeal space in a 9-year-old boy (79, 81). While these are interesting reports, they do require further medical analysis.

#### What factors may influence the assessment of the effectiveness of conservative treatment?

To properly assess the effects of treatment, a questionnaire assessing the effects should be standardised. The proposed questionnaire is presented in Table 3. The child's parents should evaluate the change in symptoms and illness, such as snoring, sleeping with the mouth open, apnoea, periods of rhinorrhoea, allergies, recurrent infections, hearing loss, or otitis media. The season in which the assessments are performed should also be taken into consideration when evaluating the effects of the treatment. Our research has shown that seasonality itself significantly affects the condition of adenoid mucus and tympanometry, but not adenoid size (46). The results of treatment should be analysed with the most objective tool; currently, the gold standard is flexible nasopharynx examination.

**Table 3:** Proposed standardised questionnaire for children suspected of adenoid hypertrophy.

First visit Date: Comments:								Control						
								Months after first visit:						
								1–3	3–6	7–9	9–12			
								The proposed conservative treatment was applied:						
								Yes			No			
								Overall improvement:						
								Yes			No			
**Season of visit**	Winter/autumn			Summer/spring				Winter/autumn			Summer/spring			
**Adenoid surgery**	yes			no				yes			no			
**Allergy**	Yes		No		Not tested			Yes		No		Not tested		
**Snoring**	Yes		Occasionally			No		Improvement		No improvement				
**Open mouth**	Yes		Occasionally		No			Improvement		No improvement				
**Hypoacusis**	Yes		Occasionally		No			Yes		Occasionally		No		
**Rhinitis**	Persistent		Seasonal			No		Improvement		No improvement				
**Rhinitis - weeks per month**	<1		1		2	3	4	<1		1	2	3	4	
**Recurrent upper respiratory tract infections**	yes				no									
**Courses of systematic antibiotics during the previous 6 months**	0	1	2	3	4	5	6	0	1	2	3	4	5	6

### Surgical adenoid treatment: adenoidectomy

Sclafani et al. reported that 90% of children with AH underwent surgery in the two-year period after the initial diagnosis (61). Slightly fewer children (70%) were operated on in Circuoli's studies regarding the effectiveness of conservative treatment of AH with intranasal beclomethasone (43). In fact, adenoidectomy is one of the most frequently performed surgeries in children (82). Bleeding is the most dangerous complications after surgery. The rate of haemorrhage following adenoidectomy is one in 200 (0.5%); taking into consideration the number of treatments performed, this affects many children. Attention should also be paid to the possibility of less frequent complications and to the child's stress associated with the first surgery. For this reason, children qualified for surgery should be well diagnosed to avoid ineffective and unjustified treatment (83). For example, adenoidectomy is recommended for the treatment of chronic rhinosinusitis in children. However, the effectiveness of adenoidectomy in chronic rhinosinusitis treatment in preschool and early-school children reaches only 47%–58% (84, 85). This could be attributed to the lack of normalised conservative treatment and appropriate diagnosis and qualification for surgery. In many cases, conservative therapy may allow time for the proper action of drugs on the adenoid and adenoid symptoms and maturation of the immunology system of the child.

Adenoid tissue regrowth after surgery may occur in 31.3% of operated children, especially those younger than five years of age (81). Such regrowth may cause a recurrence of symptoms. Some medical failures of adenoid surgery are caused by incomplete resection, whereas others can be attributed to persistent infections of the upper respiratory tract, asthma, gastroesophageal reflux (GERD), and AR (86, 87). Some of these illnesses can be diagnosed early and conservatively treated. Regrowth rate also depends on the surgeon's experience and applied surgical technique (86, 88). Yildirim showed that “blind curettage adenoidectomy” may leave up to 18% of a large residual adenoid. For total adenoid tissue resection, the nasopharynx should be controlled during the surgery by posterior rhinoscopy with the use of a mirror or trans-nasally with the use of an endoscope (88). Additionally, a study performed by Pagella et al. indicated that a greater length of the soft palate corresponds to a great risk of remnant adenoid tissue, with the authors suggesting a procedure with endoscopic control be performed, regardless of the surgical technique (89). Specifically, the authors recommended endoscopic control if the soft palate length is greater than 2.5 cm (89). The most important purpose of adenoid surgery is to precisely resect the adenoid tissue without leaving any macroscopic remnant. This increases the likelihood of resolving clinical problems related to AH. Compliance with these recommendations is expected to bring the overall rate of revision adenoidectomy down from 1.6% to 2.5% (87, 90).

## Conclusion

The diagnosis of AH should be widely based on flexible endoscopy, and other newly introduced diagnostic methods should be connected to this method. There is still no unified conservative treatment schema for AH or consensus on the length of treatment. In this respect, further research and a determination of the effects of different medical curations are indicated. Bearing in mind the fact of slow reduction of the hypertrophic adenoid under the influence of drugs, when undertaking conservative treatment, long-term therapy should be considered, with consideration of the side effects of the drugs used. The results of the treatment should be related to the most effective adenoid visualization method, which is flexible endoscopy and with the use of the Cassano and MASNA scales. Conservative therapy may be more effective when the A/C ratio remains <75%. In properly qualified patients, surgical treatment will be effective, provided that the adenoid tissue is radically resected, which is significantly more successful through intraoperative endoscopic control.
